# Redefining thoracic access: subxiphoid uniportal VATS lung resection—recovery, opioid consumption and learning curve

**DOI:** 10.3389/fsurg.2026.1922909

**Published:** 2026-09-23

**Authors:** Lubna Bakr, Filipa Pina, Hanna Binti Mohd Azhari, Tarek Khattab, Giuseppe Aresu

**Affiliations:** Department of Cardiothoracic Surgery, Royal Papworth Hospital NHS Foundation Trust, Cambridge, United Kingdom

**Keywords:** CUSUM, learning curve, lung resection, minimally invasive thoracic surgery, non-intubated thoracic surgery (NITS), subxiphoid video-assisted thoracic surgery (subxiphoid VATS)

## Abstract

**Background:**

Subxiphoid uniportal video-assisted thoracic surgery presents a more specialised approach to undertake lung resection, aiming to minimise postoperative pain. This approach has been controversial since its introduction attracting contradicting opinions from both sceptics and believers. The technique has progressed since its reported early experiences. Such controversy and progress of this technique mandates reporting the experience of one of its early adopters spanning one decade of subxiphoid VATS lung resections.

**Methods:**

Single-centre retrospective cohort study including oncological lung resections undertaken between 2016 and 2025 using subxiphoid uniportal VATS by an early adopter of this technique. Operative and perioperative outcomes were examined, including postoperative pain and opioid use. Learning curve was evaluated using cumulative sum (CUSUM) analysis. Comparative analysis evaluated outcomes before and after CUSUM inflection point.

**Results:**

126 consecutive subxiphoid VATS lung resections were included. The predominant indication was lung cancer. Median surgery duration was 127 min (IQR: 95–171). Overall conversion rate was 3.2%, and conversion to thoracotomy was required only in one patient (0.8%). Median postoperative length of stay was 3 days (IQR: 2–5). Complete R0 resection was achieved in 93.8%. There was no thirty-day mortality. 73.3% of patients with documented assessments had no pain or mild pain on postoperative day 1. Median OME was 38 mg in the first 24 h, and 15 mg between 24 and 48 h. Learning curve analysis demonstrated a significant reduction in operative time, accompanied by improvement in outcomes following the initial programme phase.

**Conclusions:**

This decade-long experience suggests that subxiphoid uniportal VATS can be performed for oncological lung resection with acceptable perioperative outcomes and low morbidity and mortality within an experienced specialist programme, with operative efficiency improving over the course of the programme. Comparative prospective studies remain necessary to establish any advantage over conventional approaches.

## 1 Introduction

Minimally invasive approaches have transformed thoracic surgery over the past few decades. The real evolution took place in the nineties when Roviaro performed the first VATS major anatomic lung resection (1). Video-assisted thoracic surgery (VATS) has since established its benefits over open thoracotomy, achieving shorter hospital stay and improved patient outcomes (2). The traditional VATS approach is performed through three ports, and this has evolved through bi-portal into uniportal approach to reduce the traumatic aspect of entry into the thoracic cavity (1). Subxiphoid video-assisted thoracic surgery (sVATS) was born as a result of a group of pioneer surgeons aiming to achieve an almost painless thoracic approach (1).

The initial sVATS experiences were reported including case reports and case series on lobectomies and segmentectomies (3–7). This technique progressed further to encompass pneumonectomies (8), opening the door to a world-first subxiphoid VATS pneumonectomy using a non-intubated, opioid-free technique performed by Aresu et al. (9, 10).

Subxiphoid VATS has progressed since its early experiences published in the literature. Patient positioning has been adjusted. Only a handful of studies have included substantial patient numbers, and to our knowledge, none has objectively quantified the impact of the approach on postoperative pain in terms of specific opioid doses or formally evaluated the learning curve. Furthermore, debate persists among both sceptics and believers regarding this technique, reflecting ongoing controversy (11, 12). Based on the available published literature, the long-term maturation of a subxiphoid VATS programme has not previously been evaluated with objective quantification of postoperative opioid consumption using weight-indexed oral morphine equivalents, or with learning curve assessment using CUSUM methodology. We hereby present this ten-year single-surgeon subxiphoid VATS lung resection experience, serving as a benchmark for future adoption and comparative studies.

## 2 Methods

### 2.1 Study design and patient population

This was a retrospective cohort study conducted at the Thoracic Surgery Unit at Royal Papworth Hospital NHS Foundation Trust. It included patients who underwent lung resection using uniportal subxiphoid VATS approach between July 2016 and December 2025 by Mr Giuseppe Aresu, one of the early adopters of this technique.

Subxiphoid VATS procedures for primary spontaneous pneumothorax (bullectomy, pleurectomy) were excluded, as these represent a distinct population with lower operative complexity and a different recovery profile. The cohort comprised all lung resections undertaken through the subxiphoid uniportal VATS approach during the study period, irrespective of the underlying pathology—predominantly primary non-small-cell lung cancer (NSCLC), together with pulmonary metastases, other primary thoracic malignancies, and a small number of diagnostic resections for indeterminate lesions—as the focus of this study is the surgical approach rather than any single histological diagnosis. Lung volume reduction surgery (LVRS) was excluded, as it constitutes a distinct population with a markedly different recovery trajectory (notably longer hospital stay and a higher rate of prolonged air leak) that would confound approach-related outcomes.

Selection of the subxiphoid approach was at the operating surgeon's discretion. Relative contraindications comprised body mass index above 30 kg/m^2^, left-sided procedures in patients with cardiac comorbidity such as cardiomegaly or arrhythmia, and reoperation. Selection criteria were not formally protocolised and evolved gradually with accumulating experience. Across the operating surgeon's overall VATS practice, subxiphoid access was used in 22.6% of procedures. Its use varied across the study period, influenced by patient selection, the availability of an experienced theatre team, and the introduction of robotic surgery to the unit.

### 2.2 Operating room set up and operative technique

The theatre set up and operative technique has progressed at our institute, and has been standardised into a smooth approach, very similar to that of uniportal VATS. Patient positioning has progressed into the standard lateral decubitus position without any inclination. Operations are undertaken through a 4-cm long longitudinal subxiphoid incision, using dedicated long subxiphoid instruments described in previous series (4, 5). The standard set-up of operating room includes the surgeon and assistant standing on the abdominal side of patient, and scrub nurse and instruments on the dorsal side. The main monitor is placed behind the patient for the use of surgeon and assistant, in addition to an additional monitor (depending on availability) on the other side allowing the scrub nurse to follow the flow of operation.

The aim behind these fine adjustments is to make this approach more friendly to regularly changing theatre staff and more reproducible to surgeons keen on adopting this technique.

### 2.3 Data collection and outcome definitions

Data were collected retrospectively from maintained paper and electronic clinical records. Baseline variables included age, sex, comorbidities, baseline haemoglobin and creatinine levels, pre-operative pulmonary function (FEV_1_ and DLCO), indication for surgery, target lobe, and extent of resection.

Operative outcomes included operative time, conversion and its causes, need for blood transfusion, and return to theatre. Postoperative outcomes included length of total and postoperative hospital stay, period requiring oxygen therapy postoperatively, drainage amount in the first 24 h, period chest drain remained *in situ*, prolonged postoperative air leak lasting more than five days, postoperative haemoglobin and creatinine, postoperative complications, and completeness of resection for oncological resections. They also included period of requiring physiotherapy, re-admission after discharge, and 30-day and 1-year mortality. One-year mortality was ascertained for patients with at least 12 months of follow-up from the date of surgery. Of 126 patients, 113 had completed 12-month follow-up at the time of data lock; the remaining 13 are excluded from the one-year mortality denominator.

From a postoperative pain perspective, outcomes included pain scores, requirement for patient-controlled analgesia (PCA), requirement for rescue IV opioids or regional blocks, as well as the oral morphine equivalents (OME) daily doses over the first 24 and 48 h postoperatively. OME doses were calculated using the relevant conversion factors for each agent, and were indexed to patient weight (13). The standard perioperative analgesic protocol consisted of multimodal analgesia including paracetamol and non-steroidal anti-inflammatory agents where not contraindicated, with patient-controlled analgesia initiated routinely in the immediate postoperative period. Rescue alternative agents, intravenous opioids, and regional nerve blocks were administered when pain was not well controlled using the above agents. The protocol evolved gradually over the study period in line with institutional enhanced recovery pathway development.

Furthermore, learning curve was examined using cumulative sum (CUSUM) analysis, and a comparative analysis evaluated outcomes before and after CUSUM inflection point.

### 2.4 Statistical analysis

Continuous variables are reported as median (interquartile range; IQR) unless stated otherwise; normally distributed data are expressed as mean ± standard deviation (SD). Categorical variables are reported as frequency and percentage. Mann–Whitney *U*-test was used for between-group comparisons for continuous variables, and chi-squared or Fisher's exact test as appropriate for categorical variables. Statistical significance was set at *p* < 0.05. Analyses were performed using Python 3.12. CUSUM analysis was performed using cumulative summation of deviations from the cohort mean operative duration. A sensitivity analysis of the learning curve was performed restricted to simple lobectomy.

Pain scores documented in four different formats were harmonised *post-hoc* into a common four-category severity framework. Sensitivity analyses were performed restricted to patients assessed using the validated 0–10 numeric rating scale, and to all patients assessed on any 0–10 numeric scale.

## 3 Results

### 3.1 Patient cohort

One hundred and twenty-six consecutive subxiphoid VATS lung resections were undertaken during the study period (Table 1). Median age was 71 years, and 46.8% were male. The majority of patients were ex-smokers (70.6%), and hypertension was the most prevalent comorbidity (42.1%), followed by COPD (31%).

**Table 1:** Baseline characteristics of the study cohort.

Variable	Total cohort (*N* = 126)
Demographics	
Age, years [median (IQR)]	71 (63–76)
Sex: Male, *n* (%)	59 (46.8%)
BMI, kg/m^2^ [median (IQR)]	24.7 (23.1–27.4)
Smoking status	
Current smoker, *n* (%)	18 (14.3%)
Ex-smoker, *n* (%)	89 (70.6%)
Never smoked, *n* (%)	18 (14.3%)
Comorbidities	
COPD, *n* (%)	39 (31.0%)
Hypertension, *n* (%)	53 (42.1%)
Diabetes mellitus, *n* (%)	14 (11.1%)
Chronic kidney disease, *n* (%)	6 (4.8%)
Pulmonary function	
FEV1, % predicted [median (IQR)]	87.5 (72.0–102.2)
TLCO/DLCO, % predicted [median (IQR)]	75.5 (56.0–88.2)
Histological diagnosis	
Primary lung cancer (NSCLC), *n* (%)	99 (78.6%)
Other primary thoracic malignancy, *n* (%)	10 (7.9%)
Pulmonary metastasis, *n* (%)	12 (9.5%)
Benign/diagnostic resection, *n* (%)	5 (4.0%)
Target lobe	
RUL (Right upper lobe), *n* (%)	28 (22.2%)
RML (Right middle lobe), *n* (%)	24 (19.0%)
RLL (Right lower lobe), *n* (%)	44 (34.9%)
LUL (Left upper lobe), *n* (%)	11 (8.7%)
LLL (Left lower lobe), *n* (%)	7 (5.6%)
Other/Multiple, *n* (%)	12 (9.5%)
Extent of resection	
Lobectomy (±synchronous procedure), *n* (%)	101 (80.2%)
Wedge resection, *n* (%)	12 (9.5%)
Segmentectomy, *n* (%)	9 (7.1%)
Bi-lobectomy, *n* (%)	1 (0.8%)
Sleeve lobectomy, *n* (%)	1 (0.8%)
Pneumonectomy, *n* (%)	2 (1.6%)
Preoperative laboratory values	
Haemoglobin, g/L [median (IQR)]	133 (124–141)
Creatinine, μmol/L [median (IQR)]	69 (60–82)

Data are presented as median (IQR) for continuous variables and *n* (%) for categorical variables. Histological diagnosis reflects final pathology; “other primary thoracic malignancy” comprises carcinoid/neuroendocrine tumours, and lymphoma. BMI, body mass index; COPD, chronic obstructive pulmonary disease; FEV1, forced expiratory volume in 1 s; TLCO, transfer factor for carbon monoxide; IQR, interquartile range; RUL/RML/RLL, right upper/middle/lower lobe; LUL/LLL, left upper/lower lobe.

Cohort characteristics are summarised in Figure 1. The predominant indication was lung cancer; on final histology the diagnosis was primary NSCLC in 78.6%, with pulmonary metastases (9.5%) and other primary thoracic malignancies (7.9%) accounting for most of the remainder, and 4% benign or diagnostic resections (Table 1). The most frequently targeted lobe was the right lower lobe in 34.9% of cases, followed by the right upper lobe in 22.2%. Lobectomy was the predominant procedure in 80.2% of cases.

**Figure 1:**
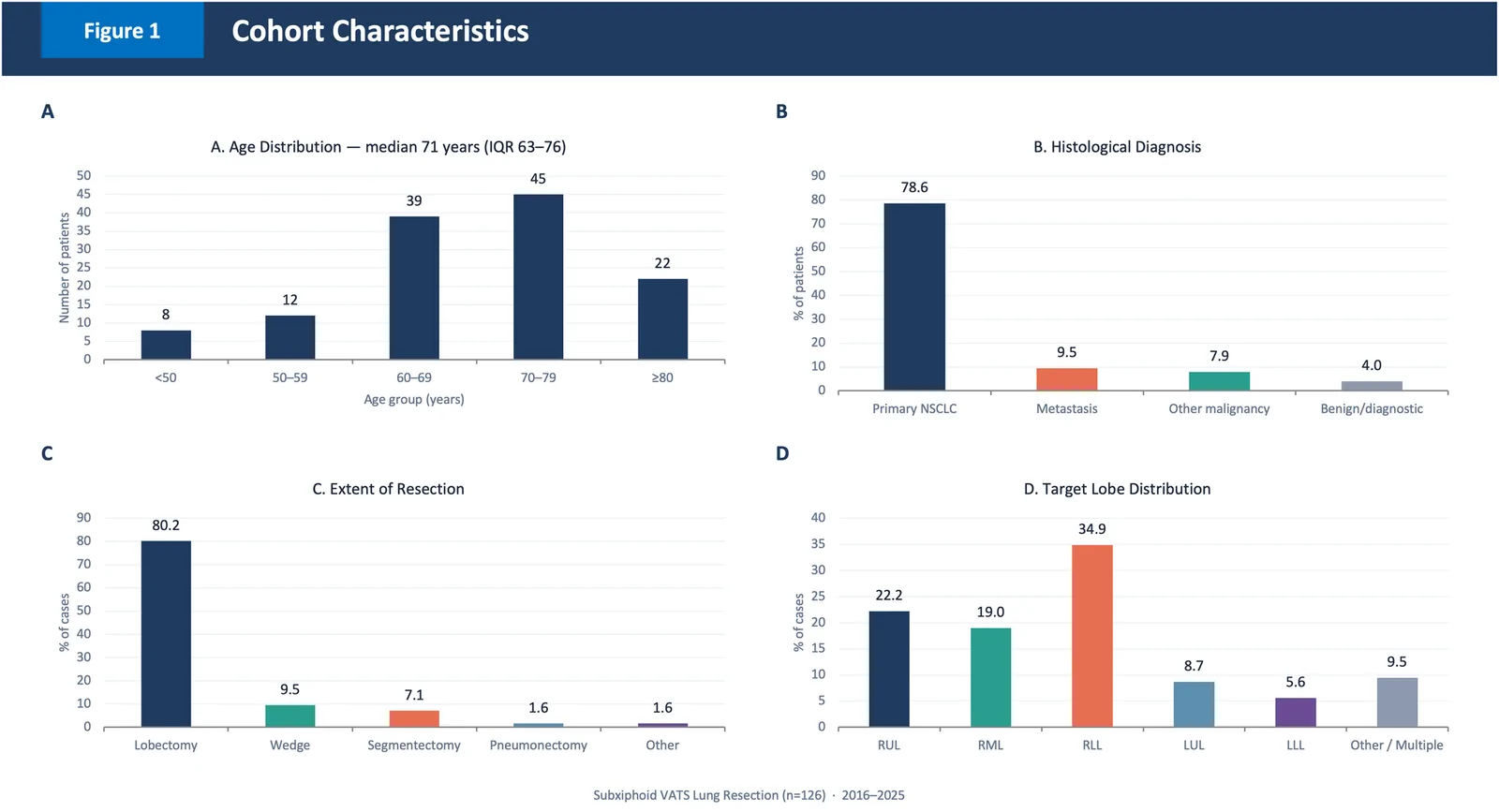
Cohort characteristics.

### 3.2 Operative outcomes

Postoperative outcomes and recovery are presented in Figure 2. Median operative time was 127 min (IQR: 95–171). Conversion was required in 4 cases only (3.2%), 3 of which required addition of intercostal VATS port to facilitate access, and only 1 required conversion to open thoracotomy due to bleeding (0.8%).

**Figure 2:**
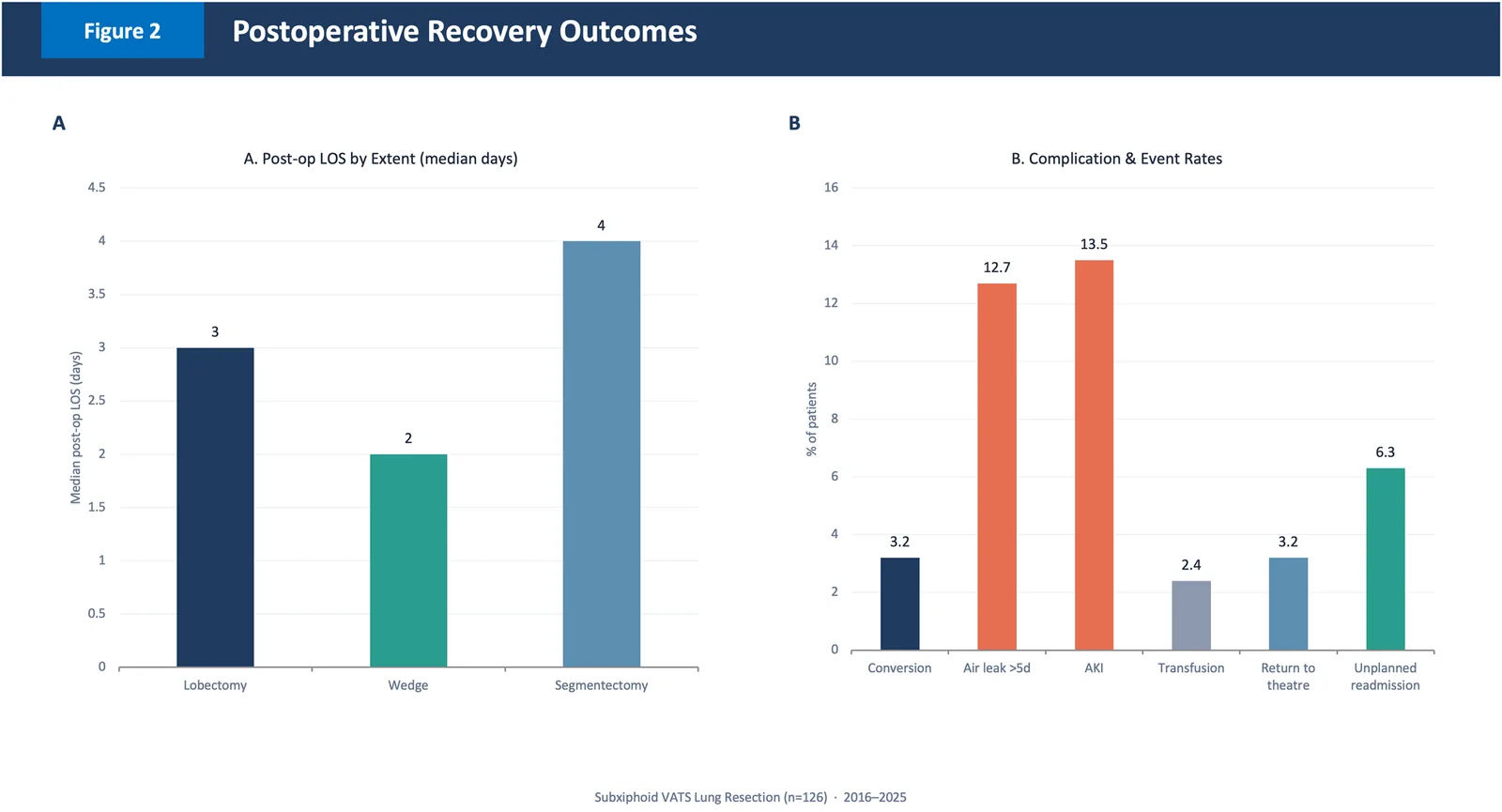
Postoperative recovery outcomes.

Seven procedures (5.6%) were performed using a non-intubated technique. Blood transfusion was required in three patients (2.4%). Four patients (3.2%) required return to theatre, 2 of which due to bleeding, 1 for middle lobe torsion following right upper lobectomy, and 1 due to non-thoracic complication to manage acute lower limb ischaemia. From oncological perspective where applicable, complete resection (R0) was achieved in 93.8%.

### 3.3 Postoperative recovery

Median postoperative length of hospital stay (LOS) was 3 days (IQR: 2–5) and total LOS was 4 days (IQR: 3–5) (Table 2).

**Table 2:** Operative and postoperative outcomes.

Variable	Total cohort (*N* = 126)
Operative	
Surgery duration (min) [median (IQR)]	127 (95–171)
Lobectomy (*n* = 95)	126 (100–160)
Lobectomy + synchronous procedure (*n* = 6)	170 (138–232)
Wedge resection (*n* = 12)	58 (52–86)
Segmentectomy (*n* = 9)	147 (110–175)
Bi-lobectomy (*n* = 1)	130
Sleeve lobectomy (*n* = 1)	240
Pneumonectomy (*n* = 2)	238 (224–252)
Non-intubated VATS, *n* (%)	7 (5.6%)
Conversion to VATS or open, *n* (%)	4 (3.2%)
Conversion to open thoracotomy, *n* (%)	1 (0.8%)
Blood transfusion, *n* (%)	3 (2.4%)
Return to theatre, *n* (%)	4 (3.2%)
Complete R0 resection (oncology subset), %	93.8%
Postoperative recovery	
Post-operative LOS (days) [median (IQR)]	3 (2–5)
Total LOS (days) [median (IQR)]	4 (3–5)
Post-op O₂ therapy (days) [median (IQR)]	0 (0–1)
Prolonged air leak (>5 days), *n* (%)	16 (12.7%)
Chest drain duration (days) [median (IQR)]	2 (1–3)
Drainage volume at 24 h (mL) [median (IQR)]	135 (62–268)
Physiotherapy duration (days) [median (IQR)]	2 (1–3)
Haemoglobin change (g/L) [mean ± SD]	−10.9 ± 10.6
Creatinine change (*μ*mol/L) [mean ± SD]	+12.7 ± 19.9
AKI, *n* (%)	17 (13.5%)
Unplanned readmission, *n* (%)	8 (6.3%)
30-day mortality, *n* (%)	0 (0%)
1-year mortality, *n* (%)	8/113 (7.1%)
Pain and opioid management	
Pain score—day 1 (standardised; *n* = 90 documented)	No pain 20 (22.2%), Mild 46 (51.1%), Moderate 19 (21.1%), Severe 5 (5.6%)
PCA initiated, *n* (%)	106 (84.1%)
Rescue IV opioid required, *n* (%)	25 (19.8%)
Rescue regional nerve block, *n* (%)	1 (0.8%)
Total OME 0–24 h (mg) [median (IQR)]	38 (18–87)
Total OME 24–48 h (mg) [median (IQR)]	15 (0–38)
Total OME 0–24 h indexed to weight (mg/kg)	0.53 (0.24–1.36)
Total OME 24–48 h indexed to weight (mg/kg)	0.24 (0.00–0.61)
OME 0–24 h vs 24–48h	*p* < 0.001
OME by extent of resection (Kruskal–Wallis)	*p* = 0.531

AKI, acute kidney injury; IQR, interquartile range; LOS, length of stay; OME, oral morphine equivalents; PCA, patient-controlled analgesia; SD, standard deviation.

Prolonged air leak (>5 days) occurred in 12.7%. Median chest drain duration was 2 days (IQR: 1–3). Median postoperative oxygen requirement was 0 days (IQR: 0–1).

Haemoglobin decreased by a mean of 10.9 ± 10.6 g/L following surgery. Creatinine increased by a mean of 12.7 ± 19.9 μmol/L, with postoperative acute kidney injury (AKI) identified in 13.5% of patients. Eight patients experienced unplanned readmission (6.3%). There was no thirty-day mortality, and one-year mortality was 7.1% (8 of 113 patients with complete 12-month follow-up).

### 3.4 Postoperative pain and opioid consumption

Pain scores on the first postoperative day were documented in 90 of 126 patients (71.4%), recorded using four formats across the study period. 32 patients were recorded using a 0–10 numeric rating scale (NRS), 15 using a 0–3 ordinal scale, 28 using raw numeric entry treated as out of 10, and 15 using categorical descriptors. To enable a unified analysis, all entries were standardised to a common four-category severity framework using pre-defined thresholds. On the 0–10 scale, 0 was classified as no pain, 1–3 as mild, 4–7 as moderate, and 8–10 as severe. On the 0–3 scale, 0 was no pain, 1 mild, 2 moderate, and 3 severe. Categorical descriptors were used directly (Figure 3).

**Figure 3:**
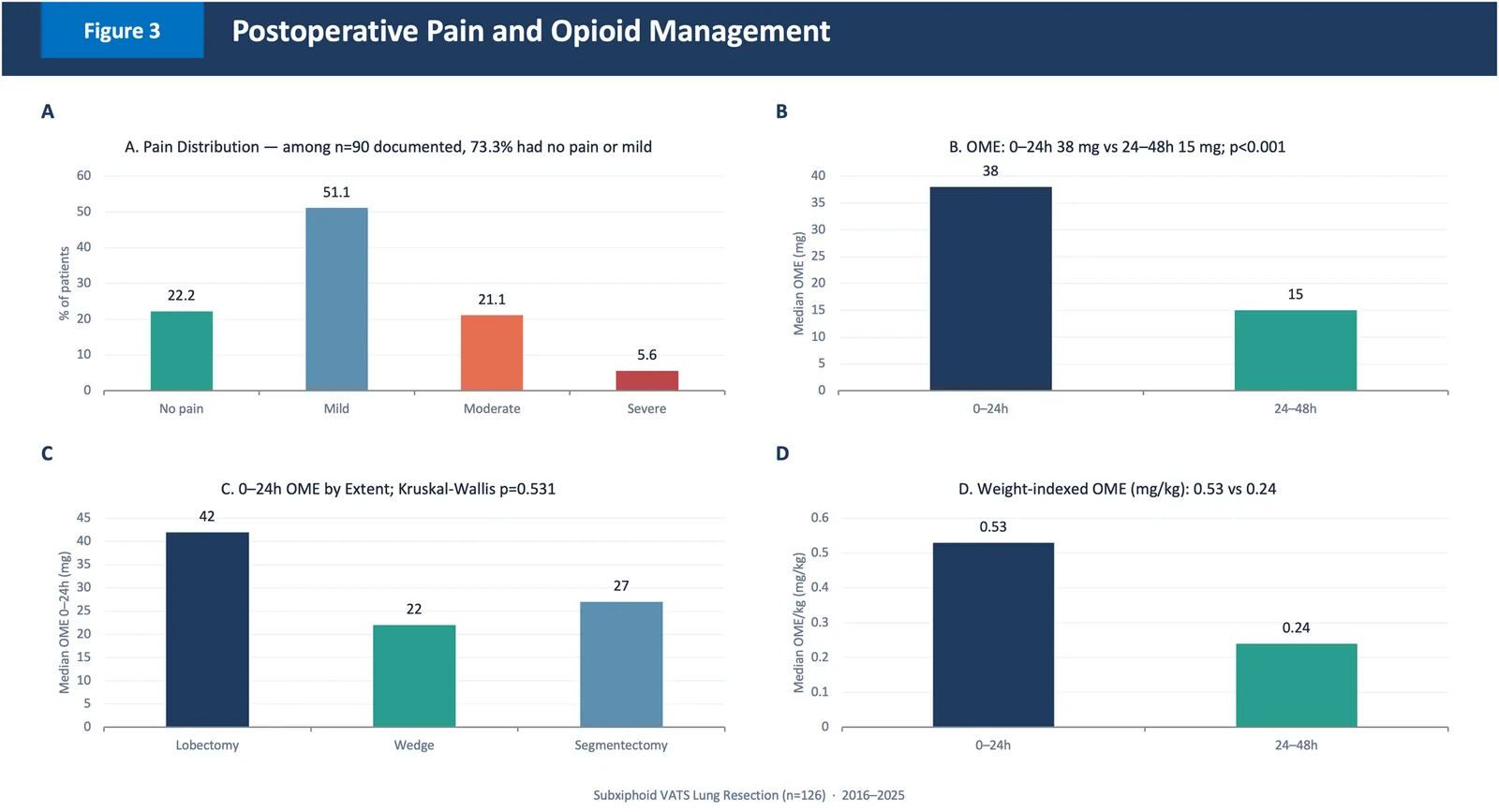
Postoperative pain and opioid management.

Following standardisation, of the 90 patients with documented scores, 20 (22.2%) reported no pain, 46 (51.1%) mild, 19 (21.1%) moderate, and 5 (5.6%) severe pain. Across all formats, 73.3% of patients with documented pain assessments reported no pain or mild pain on the first postoperative day. In a sensitivity analysis restricted to the 32 patients assessed using the validated 0–10 numeric rating scale, the median score was 2 (IQR: 0–6) and 19 patients (59.4%) reported no pain or mild pain. Across all 60 patients assessed on any 0–10 numeric scale, 42 (70%) reported no pain or mild pain.

PCA was initiated in 70% of patients reporting no pain and 95% of those with moderate pain, consistent with a pre-planned decision rather than purely reactive analgesic protocol. Rescue intravenous opioid was required in 19.8%, and rescue regional nerve block was required only in 0.8%.

Median total OME in the first postoperative 24 h was 38 mg, which more than halves to 15 mg in the 24–48 h period. This reduction in dose was statistically significant (Wilcoxon signed-rank test, *p* < 0.001). There was no significant difference in OME by extent of resection (Kruskal–Wallis *p* = 0.531).

### 3.5 Learning curve analysis

CUSUM analysis of operative duration is presented in Figure 4. The mean surgery time across the cohort was 137 min. The CUSUM curve rose progressively from the first case, reaching its peak at case 41, 36.9 months (approximately 3 years) after the first case in 2016. This identifies case 41 as the primary learning curve inflection point, marking the transition from a learning phase (cases 1–41) to an established phase characterised by sustained operative efficiency. Median surgery duration fell from 159 min pre-inflection (cases 1–41) to 110 min post-inflection (cases 42–126; Mann–Whitney *U*, *p* < 0.001).

**Figure 4:**
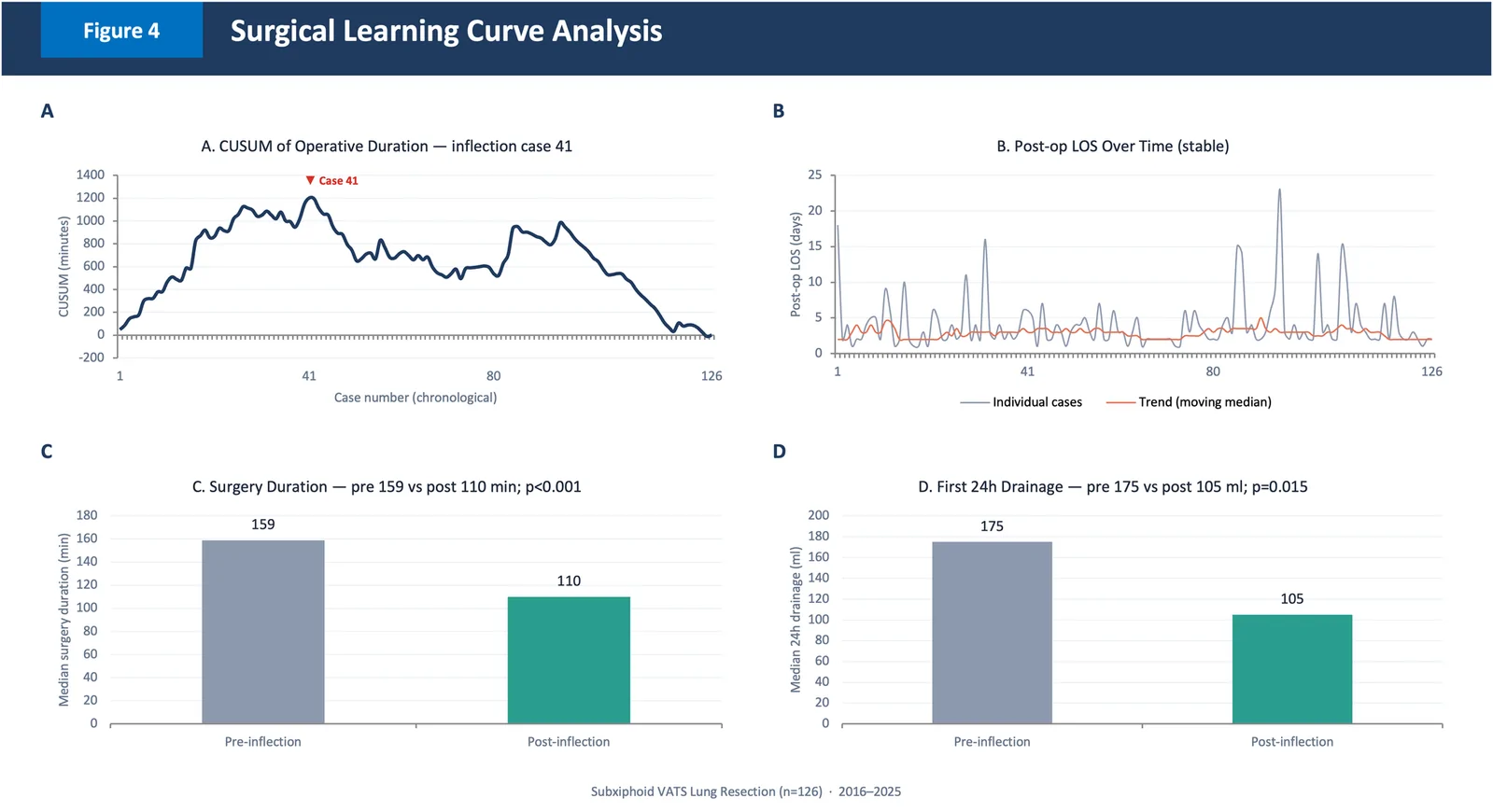
Surgical learning curve analysis.

After the primary inflection, the CUSUM curve declined overall but exhibited a transient plateau through the third quarter of the programme (cases 64–94), where median operative time rose modestly to 135 min (vs. 116 min in the second quarter). This quarter contained three of the cohort's four conversions, its highest return-to-theatre count, and the single longest procedure (369 min), reflecting a period of growth into higher procedural complexity rather than any regression in technical performance. The curve resumed its decline and returned to baseline by programme end (fourth-quarter median 100 min, with no conversions or returns to theatre).

Post-operative LOS remained stable throughout the programme, with no significant difference observed across the phases of the series. The overall conversion rate was 3.2%, with no conversions in Q2 or Q4.

### 3.6 Pre- vs. post-inflection point analysis

To further evaluate the clinical significance of the CUSUM inflection point identified at case 41, all outcomes were compared between the pre-inflection period (cases 1–41, before November 2019) and the post-inflection period (cases 42–126, after November 2019). Beyond the improvement in surgery duration (pre 159 min vs. post 110 min; Mann–Whitney *U*, *p* < 0.001), two additional outcomes improved significantly after the inflection point. AKI rate fell from 26.8% pre-inflection to 7.1% post-inflection (*p* = 0.004). Median drainage during the first postoperative 24 h came down from 175 mL (IQR: 89–350) to 105 mL (IQR: 50–200) post-inflection (*p* = 0.015). As these comparisons contrast two historical periods spanning a decade, they are reported as observed associations.

In contrast, post-operative LOS (pre 3 days vs. post 3 days; *p* = 0.93), prolonged air leak rate (17.1% vs. 10.6%; *p* = 0.392), and conversion rate (2.4% vs. 3.5%; *p* = 1.000) were not significantly different across the inflection point. No significant difference was observed in recovery or safety metrics between the two periods, although the study was not powered to exclude clinically meaningful differences in these outcomes. Unplanned readmission was likewise similar (7.3% vs. 5.9%; *p* = 0.714). The CUSUM inflection point at case 41 was therefore accompanied by improvements in renal and drainage outcomes alongside improved operative speed, with no significant difference observed in recovery or safety metrics.

### 3.7 Sensitivity analysis of the learning curve

To assess whether the observed learning curve reflected accumulating surgical experience rather than variation in case mix, a sensitivity analysis was performed restricted to simple lobectomy, the most homogeneous and most frequent procedural group in the cohort (*n* = 95).

Within this subgroup, CUSUM analysis identified an inflection point at the 67th lobectomy, 56.7 months after the first lobectomy in the series. As this analysis is based on a different case sequence and a different mean operative duration, its inflection point is not directly comparable with that of the whole cohort. The relevant finding is that the same pattern of improvement is reproduced. Median operative duration fell from 143 min (IQR: 118–178) before this point to 98 min (IQR: 89–104) after it (Mann–Whitney *U*, *p* < 0.001), and median drainage volume in the first 24 postoperative hours fell from 160 mL to 85 mL (*p* = 0.028).

The persistence of these improvements within a procedurally homogeneous group is consistent with the interpretation that they reflect accumulating operative experience rather than a shift in case complexity alone.

## 4 Discussion

This study demonstrates that the subxiphoid VATS approach has matured across the phases of the programme. This included the adjustments made in theatre set-up and patient positioning, and extended to the improvements in operative times and patient outcomes.

Early subxiphoid studies reported patient positioning in a lateral decubitus position with a 60–70° inclination (3), that later progressed to a backward inclination of 30° (4, 5). Aresu et al. has since developed this and started using the standard lateral decubitus position without any inclination. This has helped standardise the approach and make it more familiar to rotating theatre staff that do not necessarily have prior experience with sVATS. This also allows smooth conversion, if needed, by either adding an extra intercostal port(s) or proceeding to open thoracotomy. This progress has made the technique easier to adopt.

Beyond changing the inclination, several practical lessons have emerged that may assist units adopting this approach. The patient is positioned at the edge of the operating table so that instruments and camera do not clash with the table edge. Given the narrow subxiphoid corridor, the camera is placed at the inferior edge of the incision with instruments working superiorly, and bimanual instrumentation is used throughout. A 30-degree angled thoracoscope is essential, and its benefit depends heavily on an experienced assistant able to exploit the full range of angulation, as movement at the port itself is limited. Extended, double-articulated and curve-tipped instruments are required to work around the diaphragm, heart and intervening lung. Exposure can be substantially improved by placing a diaphragmatic retraction suture at entry into the pleural cavity and passing it out through the subxiphoid incision, which is of particular value in lower lobe resections. In left-sided procedures, a long curved-tip suction device can displace the pericardium medially, taking care to avoid distorting the mediastinum and compromising venous return. Our group has recently established a dedicated uniportal intercostal and subxiphoid VATS training course, aiming to support wider dissemination of this technique internationally. The aim is to overcome the reported concern regarding its reproducibility in daily practice (11).

In terms of operative factors, this study shows a conversion rate of 3.2% which compares favourably to 7% and 10.7% published in earlier subxiphoid series (4, 5). Conversion to thoracotomy was required only in 1 patient (0.8%) which is significantly less than 5.2% reported in the initial subxiphoid experience published previously in 2016 (3). Operative time, duration of chest drain, and LOS in this cohort are also improved in comparison with multiple previous subxiphoid studies (3, 14–16). Drainage volume in the 1st postoperative day is significantly less than that reported in earlier subxiphoid series (135 mL in this study vs. 180 mL in one series, 284–294 mL in another, and 595 mL in a third) (5, 14, 15). Hospital stay is comparable to a previous uniportal VATS series (17, 18), and is shorter than 7.8–9.6 days reported in earlier subxiphoid series (5). Duration of chest drain is similar to uniportal VATS (18).

From pain perspective, although the consumption of morphine in the first 24 h is higher than that reported in a previous uniportal intercostal VATS series, it is significantly lower between 24 and 48 h and is very close to those reported for uniportal VATS and less than three-portal VATS in the same series (cumulative 48 h of 53 mg in ours vs. 49.8 mg in uniportal VATS and 62.9 mg in three-portal VATS) (19). As our cohort excludes LVRS patients, it provides a directly comparable, homogeneous oncological population relative to Tosi et al, who likewise excluded severe COPD patients (19).

Although one small uniportal VATS series (15 patients) reported the median morphine dose over the first 24 h to be only 19 mg, that represented pure morphine only, not OME used in our study (18). A recently published systematic review and meta-analysis calculated the mean opioid consumption following uniportal VATS as 81.8 mg over the first 24–72 h (20). Despite the challenging direct comparison, these compare well with the doses in our study. A previously published paper suggest that the benefits of subxiphoid VATS approach extend well beyond the first 48 h, and is associated with less pain and better quality of life at 1, 3, and 6 months postoperatively (15).

Based on the available published literature, this is among the first subxiphoid VATS studies to quantify postoperative opioid consumption using specific weight-indexed OME doses, and to evaluate the subxiphoid VATS learning curve using CUSUM methodology. It therefore serves as a benchmark for future comparative studies.

Increasing operative experience may plausibly have contributed to the observed reductions in AKI and early drainage volume through more precise dissection and haemostasis. However, these comparisons span two historical periods over a decade, during which anaesthetic practice, fluid management, enhanced recovery pathways, and wider team experience all evolved. Without intraoperative haemodynamic data, these findings are best regarded as hypothesis-generating associations rather than demonstrated consequences of technical maturation.

This study has several limitations. Its retrospective, single-centre, single-surgeon design without a comparator group introduces selection bias, limits generalisability, and precludes conclusions regarding the merits of the subxiphoid approach relative to conventional uniportal intercostal VATS. Pain scores were available in 90 of 126 patients and required *post-hoc* harmonisation across four documentation formats; the proportion reporting no pain or mild pain ranged from 59.4% on the 0–10 numeric rating scale to 93.3% where categorical descriptors were used. This variation may reflect genuine differences in the populations assessed by each format, or differing thresholds between numeric and descriptive documentation. Missing pain documentation was more frequent earlier in the cohort, raising the possibility of informative missingness. As PCA was initiated in most patients, opioid consumption may partly reflect institutional analgesic practice rather than the surgical approach itself. Finally, the CUSUM analysis was based on unadjusted operative duration across procedures of differing complexity, and case 41 should be regarded as a descriptive inflection point in operative efficiency rather than a definitive proficiency threshold.

## 5 Conclusion

This study presents the maturation of the subxiphoid uniportal VATS lung resection programme across one decade. It moves the discussion beyond the believers' speech, contributing objective data to a technique that has attracted polarised opinion since its introduction. The findings suggest that subxiphoid uniportal VATS can be performed for oncological lung resection with acceptable perioperative outcomes and low morbidity and mortality within an experienced specialist programme. Operative efficiency improved over the course of the programme without measurable compromise of patient outcomes. These observations support the feasibility of the approach in specialist centres with appropriate mentorship and programme structure, while comparative prospective studies remain necessary to establish any advantage over conventional approaches.

## Data Availability

The raw data supporting the conclusions of this article will be made available by the authors, without undue reservation.
