# Supplementary material for: Redefining thoracic access: subxiphoid uniportal VATS lung resection—recovery, opioid consumption and learning curve
Source: Front Surg. 2026 Sep 23;13:1922909. doi: 10.3389/fsurg.2026.1922909 (PMC13642667; doi:10.3389/fsurg.2026.1922909)
Supplement: Supplementary file 1 [file Datasheet1.pdf]

STROBE Statement—checklist of items that should be included in reports of observational studies

	Item No.	Recommendation	Page No.	Relevant text from manuscript
**Title and abstract**	1	(*a*) Indicate the study's design with a commonly used term in the title or the abstract	1–2	Title; Abstract–Methods: retrospective cohort study
		(*b*) Provide in the abstract an informative and balanced summary of what was done and what was found	2–3	Structured abstract: Background, Methods, Results, Conclusions
**Introduction**				
Background/rationale	2	Explain the scientific background and rationale for the investigation being reported	5	Introduction: background and current controversy
Objectives	3	State specific objectives, including any prespecified hypotheses	5–6	Introduction, final paragraph: knowledge gap and study aim
**Methods**				
Study design	4	Present key elements of study design early in the paper	2, 6	Section 2.1; Abstract–Methods
Setting	5	Describe the setting, locations, and relevant dates, including periods of recruitment, exposure, follow-up, and data collection	6	Section 2.1: single centre, July 2016–December 2025
Participants	6	(*a*) *Cohort study*—Give the eligibility criteria, and the sources and methods of selection of participants. Describe methods of follow-up<br>*Case-control study*—Give the eligibility criteria, and the sources and methods of case ascertainment and control selection. Give the rationale for the choice of cases and controls<br>*Cross-sectional study*—Give the eligibility criteria, and the sources and methods of selection of participants	6–7	Section 2.1: consecutive eligible cases; exclusions and contraindications
		(*b*) *Cohort study*—For matched studies, give matching criteria and number of exposed and unexposed<br>*Case-control study*—For matched studies, give matching criteria and the number of controls per case	n/a	Not applicable
Variables	7	Clearly define all outcomes, exposures, predictors, potential confounders, and effect modifiers. Give diagnostic criteria, if applicable	7–8	Sections 2.2–2.3: baseline, operative, recovery and pain outcomes defined

Data sources/ measurement	8*	For each variable of interest, give sources of data and details of methods of assessment (measurement). Describe comparability of assessment methods if there is more than one group	7–9	Section 2.3: retrospective record review; OME conversion (ref 13); analgesic protocol
Bias	9	Describe any efforts to address potential sources of bias	6, 9, 11, 13–14, 17	Exclusions justified; sensitivity analyses; follow-up denominator; Limitations
Study size	10	Explain how the study size was arrived at	6	All consecutive eligible cases

Continued on next page

Quantitative variables	11	Explain how quantitative variables were handled in the analyses. If applicable, describe which groupings were chosen and why	9, 11	Section 2.4; Results 3.4: median (IQR) or mean ± SD; pain harmonisation thresholds
Statistical methods	12	(*a*) Describe all statistical methods, including those used to control for confounding	9	Section 2.4
		(*b*) Describe any methods used to examine subgroups and interactions	9, 11–14	Section 2.4, Results 3.4, 3.6, 3.7: OME by extent, pre/post inflection, lobectomy subgroup
		(*c*) Explain how missing data were addressed	8, 11, 17	Denominators stated; informative missingness in Limitations
		(*d*) *Cohort study*—If applicable, explain how loss to follow-up was addressed<br>*Case-control study*—If applicable, explain how matching of cases and controls was addressed<br>*Cross-sectional study*—If applicable, describe analytical methods taking account of sampling strategy	8	Section 2.3: one-year mortality restricted to 113/126 with complete follow-up
		(*e*) Describe any sensitivity analyses	9, 11, 13–14	Three sensitivity analyses: NRS-only, any 0–10 scale, simple lobectomy CUSUM
**Results**				
Participants	13*	(a) Report numbers of individuals at each stage of study—eg numbers potentially eligible, examined for eligibility, confirmed eligible, included in the study, completing follow-up, and analysed	9–10	Results 3.1: 126 consecutive procedures; no attrition
		(b) Give reasons for non-participation at each stage	6–7	No non-participation; procedure-type exclusions stated
		(c) Consider use of a flow diagram	n/a	Not required — consecutive cohort without attrition
Descriptive data	14*	(a) Give characteristics of study participants (eg demographic, clinical, social) and information on exposures and potential confounders	9–10, 21–23, Figure 1	Results 3.1; Table 1; Figure 1
		(b) Indicate number of participants with missing data for each variable of interest	8, 11, 21–23	Denominators given for pain (90/126) and one-year mortality (113/126)
		(c) *Cohort study*—Summarise follow-up time (eg, average and total amount)	8, 11	Index admission, 30 days, and 12 months (113/126 complete)
Outcome data	15*	*Cohort study*—Report numbers of outcome events or summary measures over time	10–11, 21–23, Figures 2–3	Results 3.2–3.4; Tables 1–2; Figures 2–3

		Case-control study—Report numbers in each exposure category, or summary measures of exposure	n/a	Not applicable — cohort study
		Cross-sectional study—Report numbers of outcome events or summary measures	n/a	Not applicable — cohort study
Main results	16	(*a*) Give unadjusted estimates and, if applicable, confounder-adjusted estimates and their precision (eg, 95% confidence interval). Make clear which confounders were adjusted for and why they were included	10–13, 21–23	Unadjusted medians (IQR) and n (%) with p-values throughout
		(*b*) Report category boundaries when continuous variables were categorized	11, Figure 1	Results 3.4: pain category thresholds stated; age bands in Figure 1
		(*c*) If relevant, consider translating estimates of relative risk into absolute risk for a meaningful time period	n/a	Not applicable — no relative risk estimates

Continued on next page

Other analyses	17	Report other analyses done—eg analyses of subgroups and interactions, and sensitivity analyses	11–14	Results 3.4, 3.6, 3.7: subgroup and sensitivity analyses
**Discussion**				
Key results	18	Summarise key results with reference to study objectives	14–18	Discussion; Conclusion
Limitations	19	Discuss limitations of the study, taking into account sources of potential bias or imprecision. Discuss both direction and magnitude of any potential bias	17	Limitations: design, no comparator, pain harmonisation, missingness, unadjusted CUSUM
Interpretation	20	Give a cautious overall interpretation of results considering objectives, limitations, multiplicity of analyses, results from similar studies, and other relevant evidence	16–17	Findings framed as hypothesis-generating; case 41 descriptive, not a proficiency threshold
Generalisability	21	Discuss the generalisability (external validity) of the study results	17	Single-surgeon specialist-centre setting; prospective comparative studies needed
**Other information**				
Funding	22	Give the source of funding and the role of the funders for the present study and, if applicable, for the original study on which the present article is based	18	Funding: none declared; conflicts and data availability stated

*Give information separately for cases and controls in case-control studies and, if applicable, for exposed and unexposed groups in cohort and cross-sectional studies.

Note: An Explanation and Elaboration article discusses each checklist item and gives methodological background and published examples of transparent reporting. The STROBE checklist is best used in conjunction with this article (freely available on the Web sites of PLoS Medicine at http://www.plosmedicine.org/, Annals of Internal Medicine at http://www.annals.org/, and Epidemiology at http://www.epidem.com/). Information on the STROBE Initiative is available at www.strobe-statement.org.
